# Di-μ-hydroxido-bis­[dimeth­yl(thio­cyanato-κ*N*)tin(IV)]

**DOI:** 10.1107/S1600536812043462

**Published:** 2012-11-03

**Authors:** Yaya Sow, Libasse Diop, Kieran C. Molloy, Gabriele Kociok-Köhn

**Affiliations:** a Laboratoire de Chimie Minerale et Analytique (LACHIMIA), Departement de Chimie, Faculte des Sciences et Techniques, Universite Cheikh Anta Diop, Dakar, Senegal; b Department of Chemistry, University of Bath, Bath BA2 7AY, England

## Abstract

The Sn^IV^ atom in the centrosymmetric title complex, [Sn_2_(CH_3_)_4_(NCS)_2_(OH)_2_], adopts a distorted trigonal–bipyramidal coordination environment defined by two methyl C atoms and one bridging hydroxide group in the equatorial plane while the other bridging hydroxide group and the N atom of the thio­cyanate anion are in the apical >positions. The dinuclear species are linked through O—H⋯S and C—H⋯ S hydrogen-bonding inter­actions into a three-dimensional network.

## Related literature

For background to organotin(IV) chemistry, see: Davies (2004 ▶); Gielen *et al.* (1991 ▶); Gielen (1996 ▶); Kamruddin *et al.* (1996 ▶); Khoo & Ng (2001 ▶); Tsangaris & Williams (1992 ▶). For structures containing the four-membered distannoxane [Sn(μ-OH)]_2_ unit, see: Chandrasekhar *et al.* (2007 ▶); Ng (1998 ▶). For related structures, see: Cox & Wardell (1996 ▶); Okio *et al.* (2003 ▶).
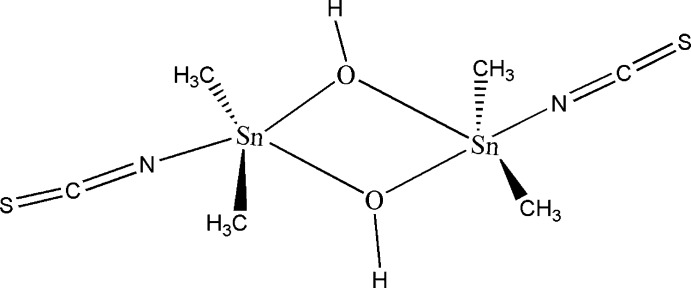


## Experimental

### 

#### Crystal data

[Sn_2_(CH_3_)_4_(NCS)_2_(OH)_2_]
*M* *_r_* = 447.69
Orthorhombic, 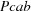

*a* = 8.3440 (2) Å
*b* = 12.5214 (3) Å
*c* = 13.3871 (2) Å
*V* = 1398.67 (5) Å^3^
*Z* = 4
Mo *K*α radiation
μ = 3.85 mm^−1^
*T* = 150 K
0.15 × 0.15 × 0.10 mm

#### Data collection

Nonius KappaCCD diffractometer
Absorption correction: multi-scan (*SORTAV*; Blessing, 1995 ▶) *T*_min_ = 0.596, *T*_max_ = 0.699
20098 measured reflections
1603 independent reflections
1333 reflections with *I* > 2σ(*I*)
*R*_int_ = 0.055

#### Refinement

*R*[*F* ^2^ > 2σ(*F* ^2^)] = 0.026
*wR*(*F* ^2^) = 0.056
*S* = 1.11
1603 reflections
71 parameters
1 restraint
H atoms treated by a mixture of independent and constrained refinement
Δρ_max_ = 1.06 e Å^−3^
Δρ_min_ = −0.64 e Å^−3^

### 

Data collection: *COLLECT* (Nonius, 1999 ▶); cell refinement: *DENZO* and *SCALEPACK* (Otwinowski & Minor, 1997 ▶); data reduction: *DENZO* and *SCALEPACK*; program(s) used to solve structure: *SIR97* (Altomare *et al.*, 1999 ▶); program(s) used to refine structure: *SHELXL97* (Sheldrick, 2008 ▶); molecular graphics: *ORTEP-3* (Farrugia, 1997 ▶); software used to prepare material for publication: *WinGX* (Farrugia, 1999 ▶).

## Supplementary Material

Crystal structure: contains datablock(s) I, global. DOI: 10.1107/S1600536812043462/wm2689sup1.cif

Structure factors: contains datablock(s) I. DOI: 10.1107/S1600536812043462/wm2689Isup2.hkl

Additional supplementary materials:  crystallographic information; 3D view; checkCIF report

## Figures and Tables

**Table 1:** Hydrogen-bond geometry (Å, °)

*D*—H⋯*A*	*D*—H	H⋯*A*	*D*⋯*A*	*D*—H⋯*A*
O—H10⋯S^i^	0.84 (2)	2.38 (2)	3.207 (3)	168 (4)
C2—H2*C*⋯S^ii^	0.98	2.79	3.746 (4)	164

Symmetry codes: (i) 
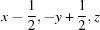
; (ii) 
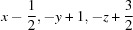
.

**Fig. 1.**
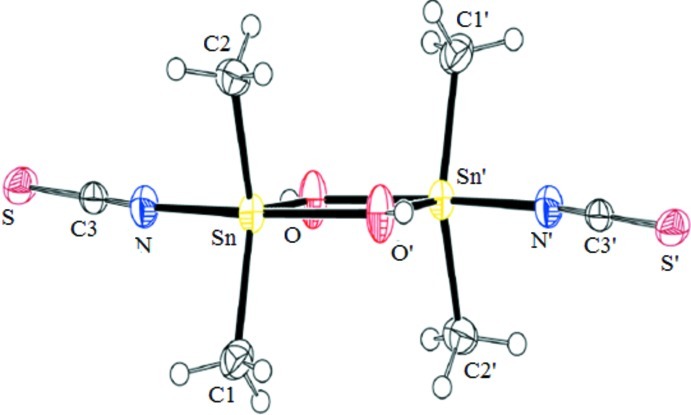
The dinuclear complex of compound (I). Displacement ellipsoids are drawn at the 50% probability level. [Symmetry code: (') -x,-y + 1, -z + 1.]

**Fig. 2.**
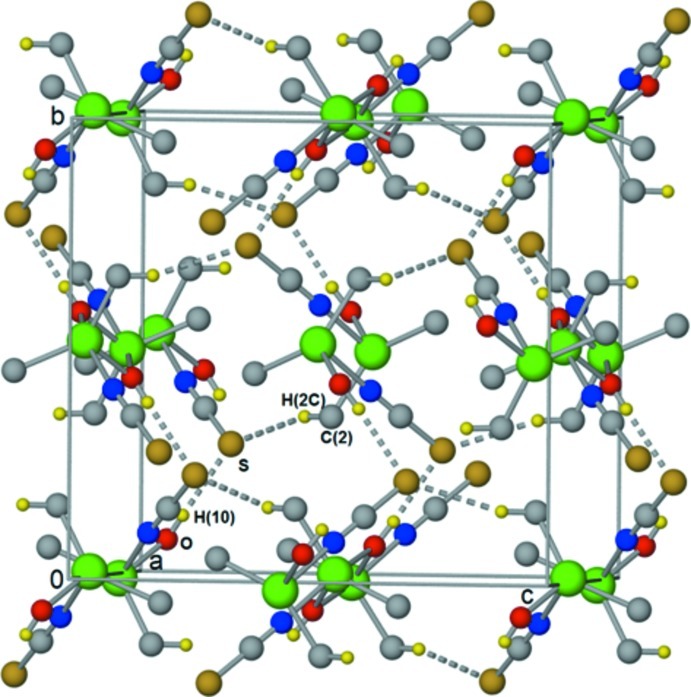
View of the hydrogen bonding system (dashed lines) between the sulfur atom of the SCN- anion and the H atom of the bridging OH group, as well as C—H···S interactions. O atoms are red, S atoms olive, N atoms blue, Sn atoms green, H atoms yellow and C atoms grey.

## supplementary crystallographic information

### Comment

Organotin complexes may interact with biological systems in many different ways as bactericides, fungicides, acaricides and industrial biocides (Davies, 2004; Gielen *et al.*, 1991; Gielen, 1996; Kamruddin *et al.*, 1996; Khoo & Ng, 2001; Tsangaris & Williams, 1992). Many tin compounds containing the Sn(CH_3_)_2_ residue tiogether with a four-membered distannoxane [Sn(µ-OH)]_2_ ring have been reported (Chandrasekhar *et al.*, 2007; Ng, 1998). In the context of new Sn(CH_3_)_2_-residue containing compounds we have initiated the structural study of the interactions between (NH_4_)SCN and Sn(CH_3_)_2_Cl_2_, which has yielded the title complex, [Sn(CH_3_)_2_(OH)(SCN)]_2_, (I).

The asymmetric unit of compound (I) is situated close to an inversion centre, which generates a dimer containing a central Sn_2_O_2_ ring; the tin(IV) atom is five-coordinated by two methyl groups, two bridging oxygen atoms and one nitrogen atom of the thiocyanate anion, forming a distorted trigonal bipyramid (Fig. 1). The sum of the angles at the tin atom, involving the carbon atoms and one O atom is 359.47 °; the nitrogen and the other oxygen atom O^i^ [(i) -*x*,-*y* + 1, -*z* + 1] are at the apical positions. The angles involving N and the atoms of the equatorial plane [N1—Sn1—C2 = 95.18 (12)°, N—Sn—C1 = 95.43 (13)°, N—Sn—O1 = 84.76 (10)°] show a significant deviation from the perfect trigonal-bipyramidal configuration. The bond lengths Sn—C [2.101 (4), 2.102 (4) Å] and Sn—N [2.220 (3) Å] are quite similar while the two Sn—O bond lengths [Sn—O1 = 2.032 (2), Sn—O^i^ = 2.198 (2) Å] are different but are in the range of typical Sn—O(bridging) distances (Ng, 1998; Chandrasekhar *et al.*, 2007). The Sn—N and Sn—C bond length are likewise in the range of reported values (Cox & Wardell, 1996; Ng, 1998; Okio *et al.*, 2003; Chandrasekhar *et al.*, 2007). The SCN^-^ anion is almost linear [N—C3—S = 178.1 (3)°], as in the structure of [(CH_3_)_4_N][Sn(C_6_H_5_)_3_(SCN)_2_] (Okio *et al.*, 2003). The Sn—N—C angle deviates more from linearity [C3—N—Sn = 172.5 (3)°].

The dinuclear species are linked through O—H···S hydrogen bonds into layers parallel to (001) (Fig. 2); C—H···S hydrogen bonding interactions (Table 1) lead to the formation of a three-dimensional network.

### Experimental

All chemicals were purchased from Aldrich (Germany) and used without any further purification. The salt (NH_4_)SCN was obtained by mixing KSCN with NH_4_Cl in ethanol 96%. The title compound (I) was synthesized by reacting Sn(CH_3_)_2_Cl_2_ with (NH_4_)SCN in ethanol (96_wt%_) in a 1:1 ratio. After stirring for two hours a clear solution was obtained that was slowly evaporated, yielding colourless crystals with a melting point of 502 K.

### Refinement

Hydrogen atoms bonded to the O atom have been located in difference Fourier maps and have been freely refined. The other hydrogen atoms have been placed onto calculated position and refined using a riding model, with C—H distances of 0.98 Å and *U*_iso_(H)= 1.5*U*_eq_(C).

### Crystal data

**Table:**

[Sn_2_(CH_3_)_4_(NCS)_2_(OH)_2_]	*F*(000) = 848
*M**_r_* = 447.69	*D*_x_ = 2.116 Mg m^−^^3^
Orthorhombic, *P**c**a**b*	Mo *K*α radiation, λ = 0.71073 Å
Hall symbol: -P 2bc 2ac	Cell parameters from 30989 reflections
*a* = 8.3440 (2) Å	θ = 2.9–27.5°
*b* = 12.5214 (3) Å	µ = 3.85 mm^−^^1^
*c* = 13.3871 (2) Å	*T* = 150 K
*V* = 1398.67 (5) Å^3^	Block, colourless
*Z* = 4	0.15 × 0.15 × 0.10 mm

### Data collection

**Table:**

Nonius KappaCCD diffractometer	1603 independent reflections
Radiation source: fine-focus sealed tube	1333 reflections with *I* > 2σ(*I*)
Graphite monochromator	*R*_int_ = 0.055
584 1.0 degree images with φ and ω scans	θ_max_ = 27.5°, θ_min_ = 4.1°
Absorption correction: multi-scan (*SORTAV*; Blessing, 1995)	*h* = −10→10
*T*_min_ = 0.596, *T*_max_ = 0.699	*k* = −16→16
20098 measured reflections	*l* = −17→17

### Refinement

**Table:**

Refinement on *F*^2^	Secondary atom site location: difference Fourier map
Least-squares matrix: full	Hydrogen site location: inferred from neighbouring sites
*R*[*F*^2^ > 2σ(*F*^2^)] = 0.026	H atoms treated by a mixture of independent and constrained refinement
*wR*(*F*^2^) = 0.056	*w* = 1/[σ^2^(*F*_o_^2^) + (0.0224*P*)^2^ + 1.8745*P*] where *P* = (*F*_o_^2^ + 2*F*_c_^2^)/3
*S* = 1.11	(Δ/σ)_max_ < 0.001
1603 reflections	Δρ_max_ = 1.06 e Å^−^^3^
71 parameters	Δρ_min_ = −0.64 e Å^−^^3^
1 restraint	Extinction correction: *SHELXL97* (Sheldrick, 2008), Fc^*^=kFc[1+0.001xFc^2^λ^3^/sin(2θ)]^-1/4^
Primary atom site location: structure-invariant direct methods	Extinction coefficient: 0.0026 (2)

### Special details

**Table:**

Geometry. All e.s.d.'s (except the e.s.d. in the dihedral angle between two l.s. planes) are estimated using the full covariance matrix. The cell e.s.d.'s are taken into account individually in the estimation of e.s.d.'s in distances, angles and torsion angles; correlations between e.s.d.'s in cell parameters are only used when they are defined by crystal symmetry. An approximate (isotropic) treatment of cell e.s.d.'s is used for estimating e.s.d.'s involving l.s. planes.
Refinement. Refinement of *F*^2^ against ALL reflections. The weighted *R*-factor *wR* and goodness of fit *S* are based on *F*^2^, conventional *R*-factors *R* are based on *F*, with *F* set to zero for negative *F*^2^. The threshold expression of *F*^2^ > σ(*F*^2^) is used only for calculating *R*-factors(gt) *etc*. and is not relevant to the choice of reflections for refinement. *R*-factors based on *F*^2^ are statistically about twice as large as those based on *F*, and *R*- factors based on ALL data will be even larger.

### Fractional atomic coordinates and isotropic or equivalent isotropic displacement parameters (Å^2^)

**Table:**

	*x*	*y*	*z*	*U*_iso_*/*U*_eq_	
Sn	0.20586 (3)	0.512859 (18)	0.486833 (17)	0.02446 (10)	
S	0.55735 (12)	0.27972 (7)	0.69604 (7)	0.0356 (2)	
O	0.0319 (3)	0.4300 (2)	0.5594 (2)	0.0365 (6)	
N	0.3739 (3)	0.4101 (2)	0.5723 (2)	0.0301 (6)	
C1	0.2597 (4)	0.4461 (3)	0.3467 (3)	0.0337 (8)	
H1A	0.1938	0.3823	0.3361	0.051*	
H1B	0.3733	0.4264	0.3445	0.051*	
H1C	0.2371	0.4985	0.2942	0.051*	
C2	0.2640 (4)	0.6541 (3)	0.5634 (3)	0.0326 (8)	
H2A	0.2317	0.7159	0.5233	0.049*	
H2B	0.3799	0.6566	0.5751	0.049*	
H2C	0.2076	0.6556	0.6276	0.049*	
C3	0.4481 (4)	0.3549 (3)	0.6243 (2)	0.0247 (7)	
H10	0.054 (4)	0.377 (2)	0.596 (2)	0.033 (10)*	

### Atomic displacement parameters (Å^2^)

**Table:**

	*U*^11^	*U*^22^	*U*^33^	*U*^12^	*U*^13^	*U*^23^
Sn	0.01938 (14)	0.02506 (15)	0.02894 (15)	0.00034 (9)	−0.00069 (9)	0.00358 (8)
S	0.0431 (5)	0.0317 (5)	0.0319 (5)	0.0115 (4)	−0.0139 (4)	−0.0056 (3)
O	0.0234 (13)	0.0358 (15)	0.0501 (16)	0.0009 (11)	−0.0028 (12)	0.0229 (12)
N	0.0249 (15)	0.0311 (16)	0.0342 (16)	0.0036 (13)	−0.0038 (13)	0.0023 (12)
C1	0.032 (2)	0.034 (2)	0.036 (2)	−0.0050 (16)	−0.0056 (15)	−0.0037 (15)
C2	0.034 (2)	0.033 (2)	0.0311 (19)	0.0002 (15)	0.0040 (15)	−0.0044 (15)
C3	0.0200 (17)	0.0275 (18)	0.0266 (16)	−0.0032 (14)	0.0030 (14)	−0.0071 (13)

### Geometric parameters (Å, º)

**Table:**

Sn—O	2.032 (2)	N—C3	1.160 (4)
Sn—C2	2.101 (4)	C1—H1A	0.9800
Sn—C1	2.102 (4)	C1—H1B	0.9800
Sn—O^i^	2.198 (2)	C1—H1C	0.9800
Sn—N	2.220 (3)	C2—H2A	0.9800
S—C3	1.625 (4)	C2—H2B	0.9800
O—Sn^i^	2.198 (2)	C2—H2C	0.9800
O—H10	0.842 (18)		
			
O—Sn—C2	111.20 (13)	Sn—C1—H1A	109.5
O—Sn—C1	112.11 (13)	Sn—C1—H1B	109.5
C2—Sn—C1	136.11 (15)	H1A—C1—H1B	109.5
O—Sn—O^i^	69.90 (10)	Sn—C1—H1C	109.5
C2—Sn—O^i^	94.13 (12)	H1A—C1—H1C	109.5
C1—Sn—O^i^	94.06 (13)	H1B—C1—H1C	109.5
O—Sn—N	84.76 (10)	Sn—C2—H2A	109.5
C2—Sn—N	95.18 (12)	Sn—C2—H2B	109.5
C1—Sn—N	95.43 (13)	H2A—C2—H2B	109.5
O^i^—Sn—N	154.66 (10)	Sn—C2—H2C	109.5
Sn—O—Sn^i^	110.10 (10)	H2A—C2—H2C	109.5
Sn—O—H10	122 (3)	H2B—C2—H2C	109.5
Sn^i^—O—H10	128 (3)	N—C3—S	178.1 (3)
C3—N—Sn	172.5 (3)		

Symmetry code: (i) −*x*, −*y*+1, −*z*+1.

### Hydrogen-bond geometry (Å, º)

**Table:**

*D*—H···*A*	*D*—H	H···*A*	*D*···*A*	*D*—H···*A*
O—H10···S^ii^	0.84 (2)	2.38 (2)	3.207 (3)	168 (4)
C2—H2*C*···S^iii^	0.98	2.79	3.746 (4)	164

Symmetry codes: (ii) *x*−1/2, −*y*+1/2, *z*; (iii) *x*−1/2, −*y*+1, −*z*+3/2.
